# *Beauveria bassiana* and *Metarhizium anisopliae* endophytically colonize cassava roots following soil drench inoculation

**DOI:** 10.1016/j.biocontrol.2016.01.002

**Published:** 2016-04

**Authors:** Melinda Greenfield, María I. Gómez-Jiménez, Viviana Ortiz, Fernando E. Vega, Matthew Kramer, Soroush Parsa

**Affiliations:** a Entomology, International Center for Tropical Agriculture (CIAT), km. 17, Recta Cali-Palmira, Colombia; b Sustainable Perennial Crops Laboratory, United States Department of Agriculture, Agricultural Research Service, Building 001, Beltsville, MD 20705, USA; c Statistics Group, United States Department of Agriculture, Agricultural Research Service, Beltsville, MD 20705, USA; d Life Sciences Innovation Center, University of California, Davis - Chile, Andrés Bello 2299 No. 1102, Providencia, Santiago, Chile

**Keywords:** *Beauveria*, Cassava, Endophyte, Fungal entomopathogen, *Metarhizium*

## Abstract

We investigated the fungal entomopathogens *Beauveria bassiana* and *Metarhizium anisopliae* to determine if endophytic colonization could be achieved in cassava. An inoculation method based on drenching the soil around cassava stem cuttings using conidial suspensions resulted in endophytic colonization of cassava roots by both entomopathogens, though neither was found in the leaves or stems of the treated cassava plants. Both fungal entomopathogens were detected more often in the proximal end of the root than in the distal end. Colonization levels of *B. bassiana* were higher when plants were sampled at 7–9 days post-inoculation (84%) compared to 47–49 days post-inoculation (40%). In contrast, the colonization levels of *M. anisopliae* remained constant from 7–9 days post-inoculation (80%) to 47–49 days post-inoculation (80%), which suggests *M. anisopliae* is better able to persist in the soil, or as an endophyte in cassava roots over time. Differences in colonization success and plant growth were found among the fungal entomopathogen treatments.

## Highlights

• First time *B. bassiana* and *M. anisopliae* introduced as endophytes in cassava roots.
• *Beauveria bassiana* and *M. anisopliae* colonized cassava roots for up to 7 weeks.
• Colonization of cassava roots by *M. anisopliae* remained relatively constant over time.
• Colonization of cassava roots by *B. bassiana* declined over time.
• Colonization levels were higher in the proximal end than in the distal end of the cassava root.

## Graphical abstract

**Figure:**
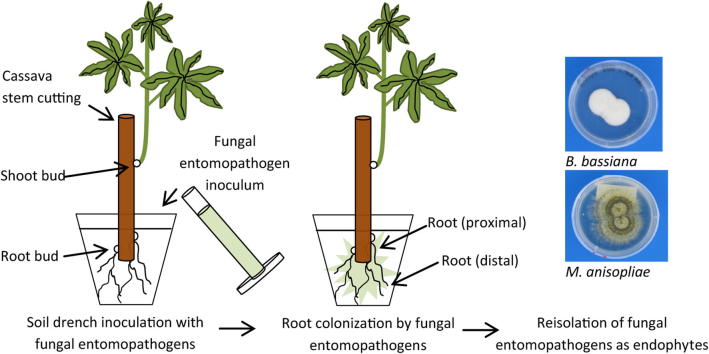

## 1 Introduction

Cassava (*Manihot esculenta* Crantz; Malpighiales: Euphorbiaceae) is the third most important food crop in the tropics after rice and maize, and is a staple food for at least 700 million people in Africa, Latin America, Asia and the Pacific Islands (Fauquet and Tohme, 2004). Cassava tolerates nutrient-poor soils and drought and is an important crop for food security and generation of income in developing countries, where it is grown mostly by smallholder farmers (Bellotti et al., 2012, Cock, 2011). The main agricultural product is the edible root, which is high in carbohydrates, but the leaves may also be eaten as a source of protein (Gleadow et al., 2009). Various parts of cassava are also used to feed livestock, and in some countries cassava starch is an important product for the paper, textiles and food processing industries (Nassar and Ortiz, 2007). Cassava is attacked by at least 200 species of arthropod pests known to cause root yield losses, including whiteflies, mealybugs, mites, hornworm, thrips and burrower bugs (Bellotti et al., 2012).

Integrated pest management (IPM) of arthropod pests in cassava includes cultural practices, host plant resistance and biological control (Bellotti et al., 2005, Bellotti et al., 2012). Cultural practices include varietal mixtures, intercropping, and the treatment of stem cuttings to ensure pest-free planting material (Bellotti et al., 2012). Host plant resistance offers an economic and environmentally sound approach but many traditional farmers use varietal mixtures in cultural practice, and it can be difficult to implement host plant resistance across multiple cassava varieties (Bellotti et al., 2012, Bellotti and Arias, 2001). Chemical pesticides can be effective for controlling many arthropod pests but are prohibitively expensive for traditional smallholder farmers, can have adverse effects on human health and the environment, and are often incompatible with IPM schemes because they can disrupt control by natural enemies (Bellotti et al., 2005, Holguín and Bellotti, 2004). Biological control using natural enemies of arthropod pests, such as predators, parasitoids and fungal entomopathogens provides an important component in IPM schemes for cassava because of its compatibility with other strategies (Bellotti et al., 2012).

Fungal entomopathogens, including *Beauveria bassiana s.l.* (Balsamo-Crivelli) Vuillemin and *Metarhizium anisopliae* (Metchnikoff) Sorokin (Ascomycota: Hypocreales), have been tested as biological control agents in laboratory and greenhouse trials against many cassava pests (Alean et al., 2004, Amnuaykanjanasin et al., 2013, Barreto et al., 2004, Jaramillo and Borgemeister, 2006, Jaramillo et al., 2005). However, when fungi are sprayed onto plants, pests can be difficult to target because of their location on the underside of leaves, such as the whitefly *Aleurotrachelus socialis* Bondar (Hemiptera: Alelyrodidae) or because they are subterranean, such as the burrower bug *Cyrtomenus bergi* Froeschner (Hemiptera: Cydnidae). The efficacy of fungal entomopathogens is also limited by abiotic factors (e.g., UV radiation, temperature, low humidity) that reduce viability of fungal conidia (Vega et al., 2012). An alternative application method is to inoculate plants with fungal entomopathogens that become established as endophytes, thereby possibly providing the plant with protection against pests from within, lowering the volume of inoculum required, and protecting the fungus against abiotic factors.

Fungal endophytes are commonly defined as fungi that live for all or part of their life cycle asymptomatically inside healthy plant tissues without causing disease (Petrini, 1991, Wilson, 1995, Hyde and Soytong, 2008). Colonization by fungal endophytes may be systemic (Gurulingappa et al., 2010, Quesada-Moraga et al., 2006), localized in plant parts (Wearn et al., 2012, Yan et al., 2015) or partitioned within plant parts (Behie et al., 2015, Zambell and White, 2014). Fungal endophytes fulfill a variety of roles comprising symbiotic and ecological functions (Rodriguez et al., 2009) that may benefit plants including improved plant growth, protection against plant pathogens (Ownley et al., 2008) and reduction of herbivory (Akello and Sikora, 2012). *B. bassiana* has been found naturally as an endophyte in several plant species and has been artificially introduced into many others (Vega, 2008). Artificial introduction of *B. bassiana* as an endophyte has been successful in maize (Bing and Lewis, 1991), cacao (Posada and Vega, 2005), date palm (Gómez-Vidal et al., 2006), coffee (Posada and Vega, 2006), banana (Akello et al., 2008), radiata pine (Brownbridge et al., 2012), fava beans (Akello and Sikora, 2012), opium poppy (Quesada-Moraga et al., 2014), cotton (Gurulingappa et al., 2010, Lopez and Sword, 2015, Ownley et al., 2008), the common bean (Parsa et al., 2013), and tomato (Ownley et al., 2008). *Metarhizium* species are less well known as endophytes but have been successfully introduced into tomato (García et al., 2011), fava bean (Akello and Sikora, 2012), oilseed rape (Batta, 2013), and haricot bean (Behie et al., 2015, Sasan and Bidochka, 2012). Two unidentified *Metarhizium* species and *M. anisopliae* have been found naturally as endophytes in roots of wall barley (*Hordeum murinum* L.) (Murphy et al., 2015).

As part of a study investigating the potential use of *B. bassiana* and *M. anisopliae* to control whiteflies (*A*. *socialis*) in cassava, we conducted greenhouse experiments to determine (1) if *B. bassiana* and *M. anisopliae* can be established as systemic or localized endophytes in cassava after soil inoculation, and (2) if inoculation with these entomopathogens affects plant growth.

## 2 Material and methods

### 2.1 Cassava plants

Cassava plants (CMC-40 variety) grown at the International Center for Tropical Agriculture (CIAT, Cali, Colombia) were used as a source of stem cuttings for all experiments. Stems of approximately 1 m length and 25 mm diameter were harvested from 9 to 10 month-old cassava plants the day before inoculations. On the day of inoculation, the 1 m stems were cut into smaller “cuttings” of approximately 200 mm in length, each with 7–9 buds. The cuttings were planted in steam-sterilized loam soil (approximately 20% clay, 50% sand and 30% silt) in disinfected pots (height 140 mm, lower diameter 105 mm, upper diameter 148 mm with approximately 1.5 kg of soil per pot) and maintained in a greenhouse with daily average (mean ± SEM) temperature of 27.5 °C ± 0.1 °C and relative humidity of 66% ± 0.3%. Each plant was fertilized with 50 mL of NPK 15:15:15 (4 g/L) 13 days after the cuttings were planted in the pots. The plants were watered as needed during experiments.

### 2.2 Fungal inoculum

The fungal inoculum was prepared following protocols modified from Parsa et al. (2013). Ten isolates/strains were used in the experiments, including five *B. bassiana* and five *M. anisopliae*. For *B. bassiana*, two isolates (CIAT 359 and CIAT 405) were obtained from the fungal entomopathogens collection at CIAT and three strains were obtained from commercially available products in Colombia, known as Beauveriplant® WP (Sanoplant, Palmira, Colombia), Bovetrópico® WP (Soluciones Microbianas del Trópico Ltda., Chinchiná, Colombia) and Micosis® WP (Bio-Protección, Chinchiná, Colombia). For *M. anisopliae*, three isolates (CIAT 001, CIAT 014A and CIAT 053) were obtained from the CIAT collection and two strains were obtained from commercially available products known as Metarhiplant® WP (Sanoplant, Palmira, Colombia) and BioMa® (Bio-Protección, Chinchiná, Colombia). Cultures of *B. bassiana* and *M. anisopliae* were grown on 75% potato dextrose agar (PDA) and oatmeal agar (Difco^TM^, Becton, Dickinson and Company, Sparks, MD) respectively, in 100 × 15 mm Petri dishes and incubated at 25 ± 2 °C with a photoperiod of 12:12 h. The cultures were allowed to grow for 14–18 days, after which conidia were harvested by scraping the surface of the agar with a sterile spatula, and rinsing the surface of the agar with sterile distilled water containing 0.1% Triton X-100. The suspensions were then filtered to remove mycelium and agar debris. Conidial concentrations were determined using an improved Neubauer haemocytometer and the suspensions were adjusted to 1 × 10^8^ conidia mL^−1^ in sterile distilled water containing 0.1% Triton X-100 to make up the required volume of inoculum for each isolate. For all experiments, conidial viability of each isolate was evaluated by taking a 100 μL sample of each inoculum, spreading it on PDA, incubating, and assessing germination 24 h later. The percentage germination of conidia was determined from 100 randomly selected conidia under a light microscope. Conidia were deemed to have germinated if hyphae were visible or the germ tube was at least twice the length of the conidia. The average of three replicate counts was calculated for each isolate.

### 2.3 Screening experiments

#### 2.3.1 Inoculation

Two screening experiments were conducted in a greenhouse to evaluate the ability of the ten isolates/strains to endophytically colonize cassava plants; one with the five *B. bassiana* isolates/strains and one with the five *M. anisopliae* isolates/strains. Each treatment consisted of 12 cassava plants, which were grown and prepared for inoculation according to the methods outlined above at 2.1. Each cassava plant root area was drenched with 100 mL of inoculum, applied to the soil surface around the base of the plant 14–15 days after the cassava cuttings were planted in the pots. At this time, the buds on the cutting of the cassava plants had already produced roots and shoots. Control plant pots were inoculated with 100 mL of sterile distilled water containing 0.1% Triton X-100. The plants were arranged in the greenhouse in a randomized block design with 12 blocks, each block containing six plants (five treatment plants and one control). A root drench was chosen as the best inoculation method after concluding a pilot study comparing root drench to immersion of cuttings. The immersion method involved the immersion of 200 mm long cassava stem cuttings in fungal inoculum for up to 2 h prior to planting the cuttings. However, this method resulted in only one root subsequently being colonized by *B. bassiana* and no roots colonized by *M. anisopliae* (unpubl. data).

#### 2.3.2 Endophyte evaluation

Due to the large number of plant samples that needed to be surface-sterilized and plated onto Petri dishes, processing for the endophyte evaluation required three consecutive days; therefore, six of the 12 blocks were evaluated for endophytic colonization by the fungal entomopathogens 7–9 days post-inoculation and the remaining six blocks were evaluated 47–49 days post-inoculation. For the first evaluation, the two longest bud roots were removed from each plant and gently washed under running tap water for approximately 30 s to remove soil particles. From each of these roots, two 60 mm pieces were taken from both the proximal and distal ends of each root (i.e., there were four 60 mm pieces of root for each plant). The root pieces were pre-washed in 0.05% Triton X-100 for three minutes and then surface-sterilized by immersing in 0.5% NaOCl (diluted in 0.05% Triton X-100) for three minutes, ethanol (70%) for one minute and then rinsing three times in sterile distilled water for 15 s each rinse. The bulk surface-sterilization system described by Greenfield et al. (2015) was used in all surface-sterilizations. To confirm that surface-sterilization was effective, eight root pieces were randomly selected from each block (with each block containing 24 root pieces in total) to make imprints on 75% PDA (in 100 mm × 15 mm Petri dishes) by gently pressing the root piece onto the surface of the agar (Schulz et al., 1998). Each root piece was then dissected into three 8 mm length sections (discarding the ends) and placed onto individual 60 mm × 15 mm Petri dishes containing PDA (75%) supplemented with antibiotics (0.1 g penicillin, 0.2 g streptomycin and 0.05 g tetracycline/L). All Petri dishes were incubated at 25 ± 2 °C in darkness and were inspected for 30 days for the presence of *B. bassiana* or *M. anisopliae*. Other fungal endophytes were also recorded and assigned morphotype codes. The proportion of root parts colonized was calculated for each plant as the number of root sections exhibiting fungal growth divided by the total number of root sections plated. The imprints were also incubated and monitored for at least 14 days for presence of fungi and if any fungi were found on an imprint, the corresponding block was discarded from the dataset.

At the 47–49 days post-inoculation evaluation, leaves and stems were evaluated as well as the roots for the presence of fungal endophytes. The roots were sampled in the same manner as described above. For the leaves, the second or third leaf (fully emerged) from the top (a young leaf) and the third leaf from the base of the plant (an old leaf) were removed from the plant. For each of these leaves, the longest lobule of the leaf was cut and trimmed to a length of 60 mm from where the leaf attaches to the petiole to the distal end of the leaf. A stem was removed from the plant and two pieces were cut from this stem; one from the top of the plant (a young stem) and one from the base of the plant where it arises from the cutting (an old stem). These stem pieces were trimmed to a length of 60 mm. Surface-sterilization proceeded as above, however the timings for leaves and stems were different from the timings used for the roots. For both leaves and stems, sterilization timings were one minute in 0.5% NaOCl, 30 s in ethanol (70%) followed by three rinses in sterile distilled water (15 s each). Imprints of leaves and stems were made as described above for roots. Leaves were dissected and six square sections (8 mm^2^) were cut from the lobule along the mid vein. Stems were dissected into approximately 8–10 mm lengths (discarding the ends). The leaf and stem sections were placed onto PDA (75%) with antibiotics (as above). Petri dishes were incubated and inspected for 30 days for the presence of *B. bassiana* or *M. anisopliae*. Other fungal endophytes were also recorded and assigned morphotype codes. The proportion of root, leaf and stem parts colonized was calculated as the number of sections exhibiting fungal outgrowth divided by the total number of sections plated.

### 2.4 Additional colonization experiments

Additional experiments were conducted with four of the best performing isolates/strains from the screening experiments (two *B. bassiana* and two *M. anisopliae*), which were selected based on the highest levels of colonization in cassava roots and the percentage of total cassava plants successfully colonized. Twelve blocks of cassava plants were inoculated in the same manner as described above for the screening experiments. Six blocks, each with five plants (the four treatment plants and one control) were evaluated for endophytic colonization by the fungi 7–9 days post-inoculation and six blocks were evaluated for endophytic colonization 47–49 days post-inoculation. The methodology for evaluating endophytic colonization was identical to that described above for the screening experiments including surface-sterilization, except for the manner in which the imprints were made after surface-sterilization. In these experiments, to confirm the effectiveness of our surface-sterilization technique, root, leaf and stem imprints were made on PDA for every individual piece of root, leaf and stem. If any fungi grew on an imprint, that individual piece of root, leaf or stem was removed from the dataset (instead of the entire block being removed).

### 2.5 Plant growth and fungal treatment differences

Various plant growth measurements were obtained from the cassava plants in both screening experiments and one of the additional (best performing isolate/strains) experiments. Aboveground measurements (stem length, number of stems, and chlorophyll content) were taken 46 days post-inoculation, i.e., the day before the plants were destroyed for the second sampling time 47–49 days post inoculation. Stem length was measured from the point of origin of the longest stem on the cutting to the tip of that stem. Leaf chlorophyll content was determined with a SPAD 502 Plus Chlorophyll Meter (Minolta Co., Ltd., Osaka, Japan). The longest lobule of the most recent fully expanded leaf was used for obtaining the SPAD value, which included an average of three measurements along the lamina of the lobule. The root measurements (dry root weight and root length) were taken over three days from 47 days to 49 days post-inoculation because they had to be measured after plants were removed from the pots. On the day of destructive sampling, the roots of each plant were collected by sieving and washed under running tap water and then dried in an oven for 72 h at 50 °C to obtain the dry root weight for each plant. The root weight was the entire root mass in the pot (minus the 2 × 60 mm pieces of root taken to evaluate endophytic colonization). The root length was the entire length of a root from where the root attached to the cassava stem cutting to the distal tip of the root (of the two longest roots).

### 2.6 Data analysis

The proportion of roots colonized by the various fungal treatments from the two screening experiments and the additional experiments were analyzed using logistic regression with random effects using the R package lme4 (Bates et al., 2015), with fungal treatment, sampling date, root part, and other endophyte presence as fixed effects and block nested in experiment as random effects. The control plant data was not included in these analyses. A means comparison, using the glht function in the R package multcomp (Hothorn et al., 2008) was used to separate the differences in proportional colonization among treatments. This method adjusts *p*-values for the number of comparisons made. A similar analysis was done using other endophyte presence as the dependent variable.

A preliminary analysis suggested that several measured plant growth variables might show differences among fungal treatments, but only the proportion of plants with roots colonized were statistically significant. Therefore we decided to create composite scores, which are often helpful in this type of analysis. The composite score can be considered a latent variable (a proxy for an unobservable dependent variable) and is a weighted linear function of the measured variables, first screened to remove those with little information value (Table 1) based on canonical (linear) discriminant analysis (Kramer et al., 2009). This method finds the optimal weighting for each measured variable’s contribution to the composite score. For these data, two orthogonal latent variables (LDA1 and LDA2) appeared adequate to describe differences in fungal treatments.

We did two analyses, using each latent variable as the dependent variable. A mixed model was estimated using the lmer function of the R package lme4 (Bates et al., 2015), with fungal treatment as a fixed effect and block nested in experiment as random effects. We used the glht function in the R package multcomp (Hothorn et al., 2008), based on a multivariate *t* distribution, to do all pairwise comparisons (method = “Tukey") of fungal treatments for each of the two composite scores.

## 3 Results

### 3.1 Screening experiments

Conidial viability was >90% for all of the *B. bassiana* and *M. anisopliae* isolates/strains in the screening experiments, except for Beauveriplant® WP, which was approximately 70% at the time of the inoculations. It is unknown why germination of Beauveriplant® WP conidia was lower for the screening experiment. All of the fungal isolates/strains in both screening experiments successfully colonized at least some cassava plant roots. At 7–9 days post-inoculation, 84% of all *B. bassiana* treated plants were colonized (including 100% of plants treated with Beauveriplant® WP and 80% treated with Bovetrópico® WP, Micosis® WP, CIAT 359 and CIAT 405), and 80% of all *M. anisopliae* treated plants were colonized (including 100% of plants treated with CIAT 014A, 75% with Metarhiplant® WP, BioMa® and CIAT 053, and 50% with CIAT 001). At 47–49 days post-inoculation, 40% of *B. bassiana* treated plants were colonized (including 67% of plants treated with Beauveriplant® WP and CIAT 359, 33% treated with Bovetrópico® WP and CIAT 405 and 0% for Micosis), and 80% of *M. anisopliae* treated plants were colonized (including 100% of plants treated with CIAT 014A, CIAT 053 and BioMa®, 80% treated with Metarhiplant® WP, and 20% with CIAT 001). *B. bassiana* and *M. anisopliae* were not found in the control plants or on any of the imprint plates. However, three imprint plates did have another (unidentified) fungus and therefore those blocks of data were removed from the analysis (one block for the *B. bassiana* screening and two blocks for the *M. anisopliae* screening). Neither *B. bassiana* nor *M. anisopliae* were found as endophytes in any leaf or stem samples.

**Fig. 1:**
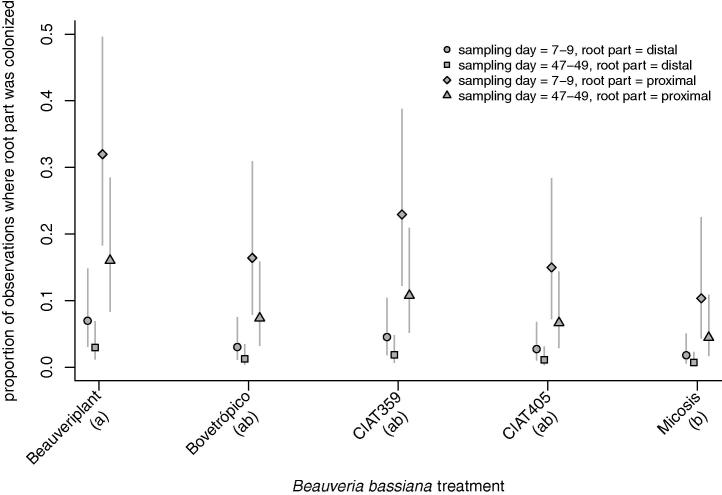
Proportion of root part colonized 7–9 and 47–49 days post-inoculation with five different *B. bassiana* isolates/strains. Root colonization levels differed significantly between Beauveriplant® WP and Micosis® WP and the highest percentage root colonization was observed for plants inoculated with Beauveriplant® WP. Colonization levels were higher in the proximal portion of the root than in the distal portion of the root and were lower at 47–49 days post-inoculation compared to 7–9 days post-inoculation. See Results section for details. The same letter underneath treatment names indicates that the means are not significantly different using Tukey’s procedure (family-wise error rate = 0.05).

**Fig. 2:**
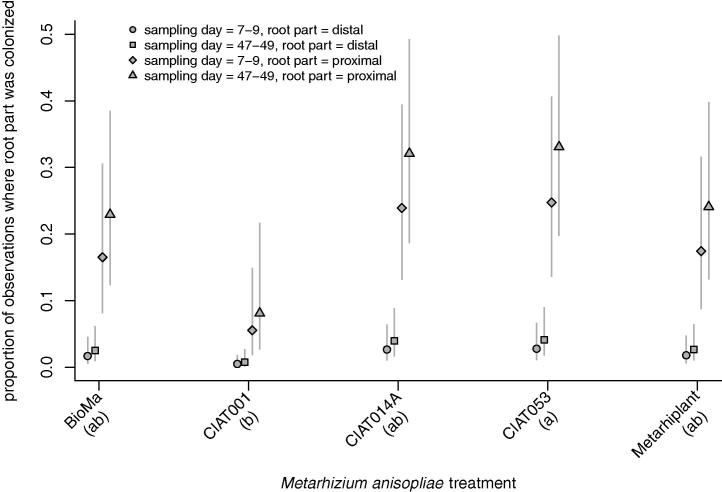
Proportion of root part colonized 7–9 and 47–49 days post-inoculation with five different *M. anisopliae* isolates/strains. Root colonization levels differed significantly between CIAT 001 and CIAT 053 and the highest percentage root colonization was observed for plants inoculated with CIAT 053. Colonization levels were higher in the proximal portion of the root than in the distal portion of the roots. The level of colonization did not vary between 7–9 days post-inoculation and 47–49 days post-inoculation. See Results section for details. The same letter underneath treatment names indicates that the means are not significantly different using Tukey’s procedure (family-wise error rate = 0.05).

Colonization levels differed significantly between some treatments in each of the screening experiments, namely Beauveriplant® WP and Micosis® WP for the *B. bassiana* screening (*χ*^2^ = 12.97, df = 4, *p* < 0.01) (Fig. 1) and CIAT 001 and CIAT 053 in the *M. anisopliae* screening (*χ*^2^ = 13.32, df = 4, *p* < 0.01) (Fig. 2). The highest percentage root colonization was observed for plants inoculated with the *B. bassiana* strain Beauveriplant® WP (Fig. 1) and the *M. anisopliae* isolate CIAT 053 (Fig. 2). Colonization levels were higher in the proximal portion of the root than in the distal portion in both the *B. bassiana* screening (Fig 1; *χ*^2^ = 42.20, df = 1, *p* < 0.001) and the *M. anisopliae* screening (Fig. 2; *χ*^2^ = 28.23, df = 1, *p* < 0.001). In the *B. bassiana* screening, colonization levels were lower at 47–49 days post-inoculation compared to 7–9 days post-inoculation (Fig. 1; *χ*^2^ = 16.22, df = 1, *p* < 0.001). In the *M. anisopliae* screening, the level of colonization did not vary between 7 and 9 days post-inoculation and 47–49 days post-inoculation (Fig. 2; *χ*^2^ = 0.11, df = 1, *p* = 0.743).

Colonization levels by other endophytes were higher at 47–49 days post-inoculation than at 7–9 days post-inoculation for both the *B. bassiana* screening (*χ*^2^ = 64.32, df = 1, *p* < 0.001) and the *M. anisopliae* screening (*χ*^2^ = 22.31, df = 1, *p* < 0.001). In the *B. bassiana* screening, there was no difference in the level of colonization by other endophytes between the proximal and distal portions. In the *M. anisopliae* screening, colonization levels by other endophytes were higher in the proximal portion than in the distal portion of the roots (*χ*^2^ = 51.62, df = 1, *p* < 0.001). The presence of other endophytes in cassava roots lowered the probability of isolating either *B. bassiana* (*χ*^2^ = 25.92, df = 1, *p* < 0.001) or *M. anisopliae* (*χ*^2^ = 62.62, df = 1, *p* < 0.001) in the screening experiments.

### 3.2 Additional colonization experiments

The four isolates/strains selected from the screening experiments for additional experimentation were CIAT 359 and Beauveriplant® WP from the *B. bassiana* screening and CIAT 014A and Metarhiplant® WP from the *M. anisopliae* screening. Metarhiplant® WP and CIAT 014A were chosen based on the 7–9 day post-inoculation evaluation. Conidial viability was >90% for all of the isolates/strains at the time of inoculation. All four isolates successfully colonized cassava plant roots with approximately 78% of *B. bassiana* treated plants and 62% of *M. anisopliae* treated plants colonized 7–9 days post-inoculation. Approximately 35% of *B. bassiana* treated plants and 67% of *M. anisopliae* treated plants were colonized 47–49 days post-inoculation. *B. bassiana* and *M. anisopliae* were not found in any control plants. Ten root imprints (from a total of 408 imprints) had fungal growth; one contained *B. bassiana* and one contained *M. anisopliae*; the rest were unidentified fungi*.* The root pieces that corresponded to these 10 imprints were removed from the dataset. *B. bassiana* and *M. anisopliae* were not found in any leaf or stem samples at any time.

Colonization levels did not differ significantly between the four fungal isolates/strains in this experiment. Colonization levels were higher in the proximal portion of the root than in the distal portion of the roots for all four *B. bassiana* and *M. anisopliae* isolates/strains (*χ*^2^ = 50.49, df = 1, *p* < 0.001). For the two *B. bassiana* isolates/strains, colonization levels were higher at 7–9 days post-inoculation compared to 47–49 days post-inoculation (*χ*^2^ = 9.23, df = 1, *p* < 0.01) and for the two *M. anisopliae* isolates/strains, the levels of colonization were higher at 47–49 days post-inoculation compared to 7–9 days post-inoculation (*χ*^2^ = 8.15, df = 1, *p* < 0.01). The levels of colonization by other endophytes did not differ significantly between the two evaluation days (i.e., 7–9 and 47–49 days post-inoculation). The presence of other endophytes lowered the probability of isolating both *B. bassiana* and *M. anisopliae* (*χ*^2^ = 34.74, df = 1, *p* < 0.001).

### 3.3 Plant growth and fungal treatment differences

We interpreted the first latent variable (1st LDA; Table 1) as colonization success. The largest contributor to it is the degree of fungal colonization of roots (for 12 root sections per plant, this is the count of those successfully colonized), with a smaller contribution from stem length and the number of roots sampled. We interpreted the second latent variable (2nd LDA; Table 1) as plant growth. The largest contributor to this variable is stem length, followed by root length and root weight, with a negative contribution from leaf chlorophyll content.

The results of multiple mean comparisons of the fungal treatments are shown in Table 2.

**Fig. 3:**
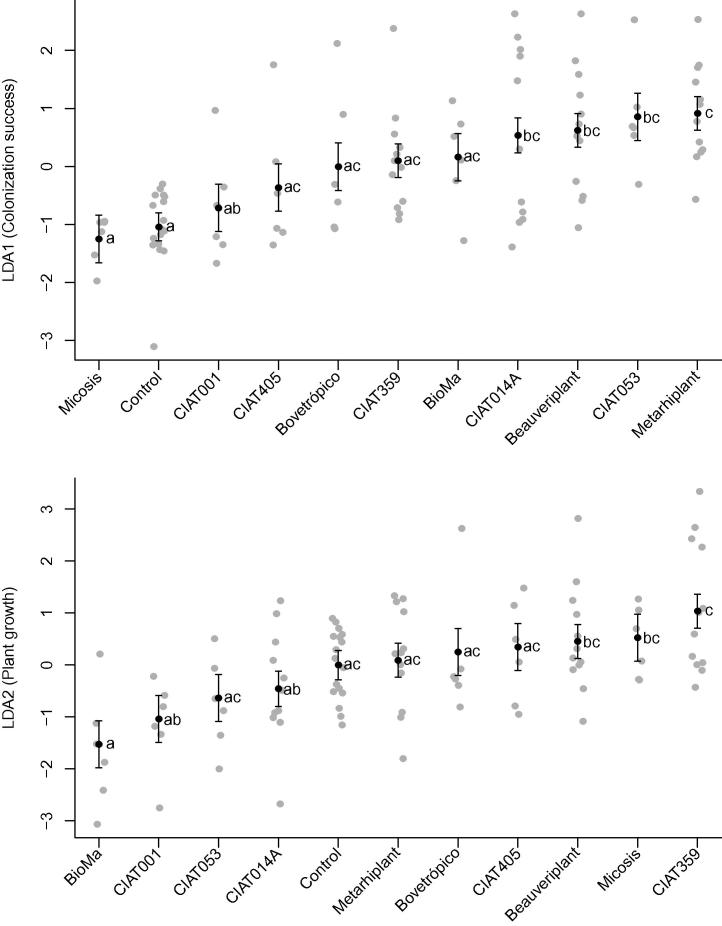
Results for the 10 fungal entomopathogens and control on the two latent axes (composite scores). LDA1 (top panel) is interpreted as colonization success, with larger numbers indicating higher success. LDA2 (bottom panel) is interpreted as plant growth, with larger numbers indicating better plant growth. Gray dots are values for each plant on their respective axes. Black dots are mixed model estimates (composite score, either LDA1 or LDA2 is the dependent variable) for each entomopathogen, with ± one standard error of the model estimate given by the vertical lines. Means separation letters follow each mean. Note that the entomopathogen order differs between panels.

The upper right triangle gives *p* values for comparisons for the first dependent latent variable (LDA1) and the lower left gives *p* values for comparisons on the second dependent latent variable (LDA2). Nine contrasts on colonization success (Fig. 3, top panel) were significant with Micosis® WP and control (similar to each other) vs Beauveriplant® WP, Metarhiplant® WP, CIAT 014A, and CIAT 053 (latter four similar to each other). Five contrasts on plant growth (Fig. 3, bottom panel) were significant with BioMa® vs CIAT 359, Beauveriplant® WP and Micosis (latter three similar to each other), and CIAT 359 vs CIAT 001 and CIAT 014A (latter two similar to each other). Metarhiplant® WP vs BioMa® was not significant (*p* = 0.0503).

## 4 Discussion

We have demonstrated for the first time that *B. bassiana* and *M. anisopliae* can endophytically colonize cassava roots. The soil drench inoculation method led to colonization of cassava roots by *B. bassiana* and *M. anisopliae* for up to seven weeks after inoculation. This suggests that successful endophytic colonization by *B. bassiana* and *M. anisopliae* can be achieved in actively growing roots of cassava. Colonization of internal plant tissues of many crops has been achieved with *B. bassiana* (Vega, 2008; see Introduction) and with *Metarhizium* species (Akello and Sikora, 2012, Batta, 2013, Behie et al., 2015) suggesting that these entomopathogens have the potential to colonize many different plant species.

We reisolated *B. bassiana* and *M. anisopliae* from surface-sterilized roots of cassava plants but never from the leaves or stems of those plants. This indicates that the fungi were not systemic within the plant, but rather remained localized in the roots. This localization is in contrast to other studies that have found *B. bassiana* can establish as an endophyte throughout the entire plant, particularly after seed inoculation (Akutse et al., 2013, Ownley et al., 2008, Quesada-Moraga et al., 2009). However, for *M. anisopliae,* our results are not surprising given that species of *Metarhizium* are more often reported as endophytes of roots and not the upper parts of plants (Akello and Sikora, 2012, Behie et al., 2015, Murphy et al., 2015). We do not know if systemic endophytic colonization by entomopathogens would be important in cassava for protecting the plant against pests of the leaves, such as the whitefly *A. socialis*. The mechanisms involved in the control of arthropod pests and diseases using endophytes include antagonism, induction of plant host defenses, host plant tolerance, or a combination of these (Ownley et al., 2010, Gómez-Vidal et al., 2009, Porras-Alfaro and Bayman, 2011). If host plant defense is induced post-inoculation with a fungal entomopathogenic endophyte, it may not be necessary for the fungus to be systemic (Jaber and Vidal, 2010). In the present study, an attempt to investigate the effects of the fungal treatments on resistance by cassava to *A. socialis* was made, but large variability in the results among replicates precluded learning whether the various endophytic fungal entomopathogens differentially affected resistance by cassava against insects (unpubl. data).

There are several possible explanations for the lack of systemic colonization by our isolates/strains. Firstly, some studies have shown that colonization by the applied fungus is more likely in the plant part that was in direct contact with the inoculum and less likely or not at all in plant parts distant to the application site (Akello et al., 2007, Akello et al., 2009, Tefera and Vidal, 2009). This would explain why our soil drench inoculation resulted in colonization only in the roots. This is supported by several surveys that have suggested there is a lack of evidence for systemic growth by fungal endophytes from one plant tissue type to another (Wearn et al., 2012, Yan et al., 2015). Secondly, competition with other endophytes is likely to be important. Indeed, approximately 40 other morphospecies were recovered from the surface-sterilized root samples in our study and our analyses showed that the probability of finding *B. bassiana* and *M. anisopliae* was reduced significantly when other endophytes were present. We do not know if these other endophytes originated from the environment in which the plants were growing or from the stem cutting itself. The stem cutting may contain a store of fungal and bacterial endophytes that originate from the parent plant and these could compete with *B. bassiana* and *M. anisopliae* inside the plant. A study investigating the endophyte community within the stem cutting and how these other endophytes interact with *B. bassiana* and *M. anisopliae* would be useful.

Colonization by *B. bassiana* and *M. anisopliae* was higher in the proximal portion of the root than in the distal portion across all of our experiments. It is unknown if conidia of these fungi were concentrated in the upper soil strata, where the proximal end of the root is located but this is one hypothesis that could explain our results (Kim et al., 2010, Storey and Gardner, 1988). Future studies could evaluate the presence of fungal entomopathogens inoculated into the soil in the different soil strata to determine if conidia adhere to soil particles in the upper soil layer around cassava stem cuttings. Another hypothesis is that the proximal end of the root provides different conditions that influence colonization. For example, in some cassava varieties, the proximal end of cassava root has been found to contain higher levels of cyanogenic glycosides than the distal end (Cooke, 1978). We do not know if this is the case in the cassava CMC-40 variety used in our experiments, which is low in cyanogenic glycosides overall, but it shows that conditions can be different across the longitudinal gradient of cassava roots.

*B. bassiana* and *M. anisopliae* persisted in cassava roots for up to seven weeks in all of the experiments. *M. anisopliae* colonization levels remained relatively constant over time (in the screening experiment) and increased in time (for the additional experiment) between the two sampling dates, whereas *B. bassiana* colonization levels decreased by half between sampling dates in all experiments. This is not surprising given previous studies have shown *M. anisopliae* is rhizosphere competent (Bruck, 2005, Bruck, 2010, Hu and St. Leger, 2002, St. Leger, 2008) and persists well in the soil environment, but *B. bassiana* does not persist as well (Lingg and Donaldson, 1981, Vänninen et al., 2000). Further, *B. bassiana* is more commonly found aboveground whereas *M. anisopliae* is very common belowground (Meyling et al., 2011). At the same time, the presence of other endophytes increased over time (screening experiments) or remained constant (additional experiment) and, as mentioned above, for those plants that had other endophytes, the probability of finding either *B. bassiana* or *M. anisopliae* was reduced. This indicates that the presence of other endophytes in cassava roots influenced the levels of colonization by both *B. bassiana* and *M. anisopliae*. The reason that the level of colonization by *M. anisopliae* remained constant despite an increase in other endophytes is likely to be related to its competence in the rhizosphere. There was opportunity for other endophytes to increase over time because at both sampling times, some cassava root pieces were not colonized by any fungi. In other words, colonization by other endophytes could increase over time despite the level of colonization by *M. anisopliae* being maintained and this is evident because there were less un-colonized root pieces overall at the second sampling time.

Using the composite scores we have shown clear differences in the fungal entomopathogen isolates/strains in terms of colonization success and plant growth. These results show that the commercial products are not equivalent, with one of them (Micosis® WP) being no different than the control on the first composite score. Two of the entomopathogens (CIAT 053 and Beauveriplant® WP) were among the best performing for colonization success and two (CIAT 359 and Beauveriplant® WP) were among the best for plant growth. Only one of the 10 fungal entomopathogens (Beauveriplant® WP) resulted in both higher colonization success and plant growth, indicating it would be a good candidate for further studies. The negative loading for chlorophyll was unexpected; it could be a result of increased plant resource allocation to stem and roots, consequently not producing as much chlorophyll.

Our results support previous studies where plant growth promotion has been reported for *B. bassiana* (Lopez and Sword, 2015) and *M. anisopliae* (Kabaluk and Ericsson, 2007). Future research could allow inoculated cassava plants to grow for a longer period of time to investigate the influence of entomopathogens as endophytes on plant growth and whether root growth in particular increases, which might be beneficial for increasing root yield.

## Figures and Tables

**Table 1:** Linear discriminant analysis (LDA) weights (loadings) for variables including plant growth measurements and degree of colonization by fungi. These were used to create the composite scores used for statistical comparisons of the treatment fungi. Variables were scaled to mean = 0, standard deviation = 1 prior to calculating weights.

Variables	Weight (1st LDA)	Weight (2nd LDA)
Root (dry weight total)	−0.265	0.267
Root length	0.223	0.337
Number of roots sampled	0.323	−0.129
Leaf chlorophyll content (SPAD value)	0.029	−0.480
Stem length	0.411	0.956
Number of stems on plant	−0.122	0.152
Degree of fungal colonization of roots	1.129	−0.032

**Table 2:** *p*-values for *a posteriori* pairwise comparisons of composite scores. Composite scores were created using predictions from the first two dimensions of a linear discriminant analysis using six cassava plant health measurements (root dry weight, root length, number of roots sampled, stem length, number of stems on plant, leaf chlorophyll content) and degree of colonization of cassava roots by treatment fungi. The upper right portion of the table is for LDA1 and the lower left portion LDA2.

Bb = *Beauveria bassiana*

Ma = *Metarhizium anisopliae*
